# Comparison between clinical features and prognosis of malignancy- and non-malignancy–associated pediatric hemophagocytic lymphohistiocytosis

**DOI:** 10.1186/s12887-019-1702-5

**Published:** 2019-11-29

**Authors:** Hua Pan, Yongmin Huo, Lirong Sun

**Affiliations:** 1 Department of Paediatric Hematology, Affiliated Hospital of Qingdao University, 16 Jiangsu Road, Qingdao, 266003 Shandong China; 2 Department of Paediatrics, Women and Children’s Health Care Hospital of Linyi, 187 Qiyang Road, Linyi, 276000 Shandong China

**Keywords:** Hemophagocytic lymphohistiocytosis, Hematopoietic stem cell transplantation, Perforin, Child

## Abstract

**Background:**

The differences between the clinical characteristics and survival time in malignancy- and non-malignancy–associated secondary hemophagocytic lymphohistiocytosis (HLH) are unclear. Here, we describe the clinical characteristics, prognostic factors, and survival outcomes of malignancy-associated HLH compared to that of non-malignancy–associated HLH.

**Methods:**

We retrospectively analyzed 91 pediatric patients with HLH (age < 14 years) at the Affiliated Hospital of Qingdao University Pediatric Department between January 2005 and October 2016. The patients were divided into the malignancy-associated group (*n* = 22) and non-malignancy–associated group (*n* = 69, also considered the control group). The clinical features were compared using the Mann–Whitney U and χ^2^ tests. The overall survival time was compared using log rank and Mann–Whitney U tests.

**Results:**

Hemoglobin (HGB; *p* = 0.004), alanine aminotransferase (ALT; *p* = 0.002), and aspartate aminotransferase (AST; *p* = 0.001) levels in the malignancy-associated group differed from that in the non-malignancy–associated group. The mean survival times were 26.9 ± 3.82 months (malignancy-associated HLH) and 35.03 ± 2.19 months (non-malignancy–associated HLH). The overall survival time between the two groups was not statistically significantly different (*p* = 0.055). Univariate analysis showed that disseminated intravascular coagulation (DIC) score > 5 (*p* = 0.001), albumin < 25 g/L (*p* = 0.000), HGB < 60 g/L (*p* = 0.001), and platelet count (PLT) < 30 × 10^9^/L (*p* = 0.042) correlated with prognosis. Multivariate Cox analysis showed that albumin < 25 g/L (*p* = 0.017), HGB < 60 g/L (*p* = 0.027), and bone marrow hemophagocytosis (*p* = 0.034) correlated with worse prognosis.

**Conclusions:**

Patients with non-malignancy–associated HLH do not have better survival, although their prognosis is relatively better in clinical practice. A higher DIC score at diagnosis and lower albumin, HGB, and PLT levels are negative prognostic factors in malignancy-associated HLH.

## Background

Hemophagocytic lymphohistiocytosis (HLH) is characterized by aberrant activation and proliferation of polyclonal CD8^+^ T lymphocytes and macrophages that infiltrate multiple organs and overproduce inflammatory cytokines [1]. HLH has been characterized as primary HLH or secondary HLH (SHLH). Primary HLH is often associated with inherited gene defects.

SHLH generally occurs later in life than primary HLH. SHLH is an acquired phenomenon that develops in response to severe infections, autoimmune or rheumatologic disorders, drugs, and in association with concurrent malignancies [2]. Malignancy-associated HLH is most commonly seen in acute leukemia in children. HLH can occur as initial HLH or concomitantly with malignant disease. The mechanism of malignancy-associated hemophagocytosis is not well understood, and may be associated with cytokine secretion (including interferon-γ and interleukin-6), persistent antigen stimulation by malignant cells, chemotherapy-induced immunosuppression, hematopoietic stem cell transplantation (HSCT), or infection [3].

The difference between malignancy- and non-malignancy–associated (i.e., causes other than tumor) HLH remains unclear. We retrospectively reviewed patients with malignancy- and non-malignancy–associated HLH at our institution and compared the clinical characteristics, treatment response, overall survival time, and prognostic factors of the two groups. In this study, we also share our limited experiences with allogeneic HSCT in malignancy-associated HLH. We aimed to distinguish between malignancy-associated and non-malignancy–associated HLH, compare their clinical features and prognostic significance, and analyze the difference between the two conditions.

## Methods

### Participants

The clinical data of 91 patients aged < 14 years who had been diagnosed with HLH from January 2005 to October 2016 at the Children Medical Center of the Affiliated Hospital of Qingdao University were included and retrospectively reviewed after we had received University of Qingdao Ethics Committee approval. A retrospective historical cohort study was carried out. Primary HLH was excluded.

Patients were diagnosed with HLH according to HLH-2004 diagnostic guidelines [4] and were divided into malignancy-associated HLH (*n* = 22; 13 boys and nine girls) and non-malignancy–associated HLH (*n* = 69; 30 boys and 39 girls) groups for improved comparison of the clinical features and outcome. The data collected for each patient included age, sex, presence or absence of splenomegaly, neutrophils (absolute neutrophil count, ANC), hemoglobin (HGB), platelet (PLT), Epstein–Barr virus (EBV) DNA copy number, aspartate aminotransferase (AST), alanine aminotransferase (ALT), lactate dehydrogenase (LDH), albumin, presence of jaundice, presence of cholecystitis, triglycerides (TGs), fibrinogen (Fib), ferritin, disseminated intravascular coagulation (DIC) score, and bone marrow hemophagocytosis. The DIC score at admission was calculated in accordance with International Society of Thrombosis Hemostasis criteria [5].

### Statistical analysis

The clinical features of the two groups were compared using Mann–Whitney U tests for skewed continuous data, and with χ^2^ tests for categorical data. All results are presented as the median and range (min–max) as indicated. Overall survival time, defined as the time from HLH diagnosis to death from any cause, was estimated with the Kaplan–Meier method. The overall survival time of the two groups was compared using log rank and Mann–Whitney U tests. The relationship of prognostic factors associated with malignancy-associated HLH was evaluated using Pearson correlation for two normally distributed variables and with Spearman rank correlation for non-normally distributed variables. Prognostic factors associated with malignancy-associated HLH were evaluated using univariate and multivariate Cox proportional hazard models. Multiple group corrections were performed using the false discover rate. In all tests, *p* < 0.05 was considered significant. All statistical analyses were performed using SPSS version 13.

## Results

### Clinical characteristics

There was no significant difference between age, sex, splenomegaly, ANC, PLT, presence of EBV infection, albumin, presence of jaundice, and presence of cholecystitis in the two groups (Table 1). Serum TG, ferritin, Fib, and LDH levels and presence of DIC were also not significantly different between the two groups. However, patients with non-malignancy–associated HLH had significantly higher HGB (*p* = 0.004), ALT (*p* = 0.002), and AST (*p* = 0.001) levels than the patients with malignancy-associated HLH.

**Table 1:** Clinical features of malignancy-associated hemophagocytic lymphohistiocytosis (M-HLH) and non-malignancy–associated hemophagocytic lymphohistiocytosis (N-M-HLH)

	M-HLH (*n* = 22)	N-M-HLH (*n* = 69)	*Statistic value*	*p*-value	*Q* value
Age [year; median (min, max)]	3.60(0.25,13.00)	1.20(0.20,13.00)	−2.216^†^	0.027^b^	0.135
Sex					
Male	13.00(59.10%)	39.00(56.50%)	1.111*	0.045^a^	0.113
Female	9.00(40.90%)	30.00(43.50%)			
Splenomegaly					
No	3.00(13.60%)	22.00(31.90%)	2.965*	0.095^a^	0.211
Yes	19.00(86.40%)	47.00(68.10%)			
Neutrophils [10^9^/L; median (min, max)]	1.04(0.05,8.00)	1.10 (0.05,10.50)	−0.496^†^	0.620^b^	0.775
Hemoglobin [g/dL; median (min, max)]	69.50(54.00,115.00)	85.00 (31.00,131.00)	−2.879^†^	0.004^b^	0.027
Platelets [10^9^/L; median (min, max)]	43.50(2.00,293.00)	62.00 (7.00,418.00)	−2.077^†^	0.038^b^	0.109
TG [mg/dL; median (min, max)] ()	3.13(0.81,7.55)	3.42 (0.05,13.56)	−0.385^†^	0.700^b^	0.778
Fibrinogen [mg/dL median (min, max)]	1.24(0.58,5.00)	1.20 (0.27,4.56)	−0.959^†^	0.337^b^	0.518
Ferritin [mg/dL; median (min, max)]	1148.50(354.00,21,173.00)	3562.00 (132.00,27,810.00)	−0.742^†^	0.458^b^	0.654
LDH [IU/L; median (min, max)]	1002.50(227.00,17,958.00)	936.00 (156.00,5311.00)	−0.649^†^	0.516^b^	0.688
DIC					
No	18.00(81.80%)	44.00(63.80%)	0.391*	0.114^a^	0.228
Yes	4.00(18.20%)	25.00(36.20%)			
Jaundice					
No	14.00(63.60%)	40.00(58.00%)	0.788*	0.638^a^	0.751
Yes	8.00(36.40%)	29.00(42.00%)			
Hemophagocytosis					
Yes	16.00(72.70%)	37.00(53.60%)	2.306*	0.114^a^	0.207
No	6.00(27.30%)	32.00(46.40%)			
EBV					
Positive (EBV DNA > 1.44 × 10^3^/copies)	8.00(36.30%)	21.00(30.40%)	1.306*	0.037^a^	0.123
Negative (EBV DNA < 1.44 × 10^3^/copies)	14.00(63.70%)	48.00(69.60%)			
ALT [g/L; median (min, max)]	77.50(17.00,403.00)	210.00(3.00,5679.00)	−3.110^†^	0.002^b^	0.02
AST [g/L; median (min, max)]	87.50(19.00,320.00)	210.00(12.00,7654.00)	−3.407^†^	0.001^b^	0.02
Albumin [g/L; median (min, max)]	30.00(18.80,37.60)	29.00 (17.91,40.30)	−0.269^†^	0.788^b^	0.829
Cholecystitis					
Yes	1.00(4.5%)	10.00(14.5%)	0.281*	0.213^a^	0.355
No	21.00(95.5%)	59.00(85.5%)			
Pneumonia					
Yes	10.00(45.50%)	32.00(46.40%)	0.964*	0.940^a^	0.940
No	12.00(54.50%)	37.00(53.60%)			
Pleural effusion					
Yes	3.00(13.60%)	26.00(37.70%)	0.261*	0.035^a^	0.140
No	19.00(86.40%)	43.00(62.30%)			

*TG* Triglyceride, *LDH* Lactate dehydrogenase, *DIC* Disseminated intravascular coagulation, *HLH* Hemophagocytic lymphohistiocytosis

Q-value: *P* value after multiple group corrections

^a^*p*-value for χ^2^ test; *: Odds Ratio for Chi-square test

^b^*p*-value for Mann–Whitney U test; †: Z value for Mann–Whitney U test

### Malignancy-associated HLH

The median (range) age of patients in the malignancy-associated HLH group was 3.60 (0.25–13.00) years. The most frequent histological subtype was leukemia, which was diagnosed in 17 of the 22 patients (77.3%); the remaining five patients (22.7%) had lymphoma [Hodgkin lymphoma (HD), *n* = 2; non-HD, *n* = 3)]. Of the 17 children with leukemia, nine had acute lymphoblastic leukemia (ALL; B-ALL, *n* = 5; T-ALL, *n* = 4) and eight had AML.

Thirteen patients (59.1%) were treated according to HLH-2004 as initial therapy for HLH, and four patients received chemotherapy as initial therapy. The overall response rate was 68.2% (complete remission rate, 40.9%; partial remission rate, 27.3%).

### Non-malignancy–associated HLH

The median (range) age of patients in the non-malignancy–associated HLH group was 1.20 (0.20–13.00) years. Of the 69 patients in the group, 21 (30.4%) had EBV-associated HLH, 10 (14.5%) had cytomegalovirus infection, 10 (14.5%) had bacterial infections, nine (13.0%) had immunodeficiency diseases (chronic granulomatous diseases, *n* = 4; congenital disorder of fucosylation, *n* = 3; common variable immunodeficiencies, *n* = 2), five (7.2%) had chronic active EBV (CAEV) infection, five (7.2%) had autoimmune disease (systemic lupus erythematosus [SLE], *n* = 4; Still’s disease, *n* = 1), two (2.9%) had fungal infections, and seven (10.1%) had other diseases.

Forty-one patients (59.4%) were treated according to HLH-2004 as initial therapy for HLH, 18 patients (26.1%) received high-dose immunoglobulin and glucocorticoid chemotherapy, and 10 patients (14.5%) received glucocorticoids only as initial therapy. The overall response rate was 75.6% (complete remission rate, 55.1%; partial remission rate, 20.5%).

### Outcome

**Fig. 1:**
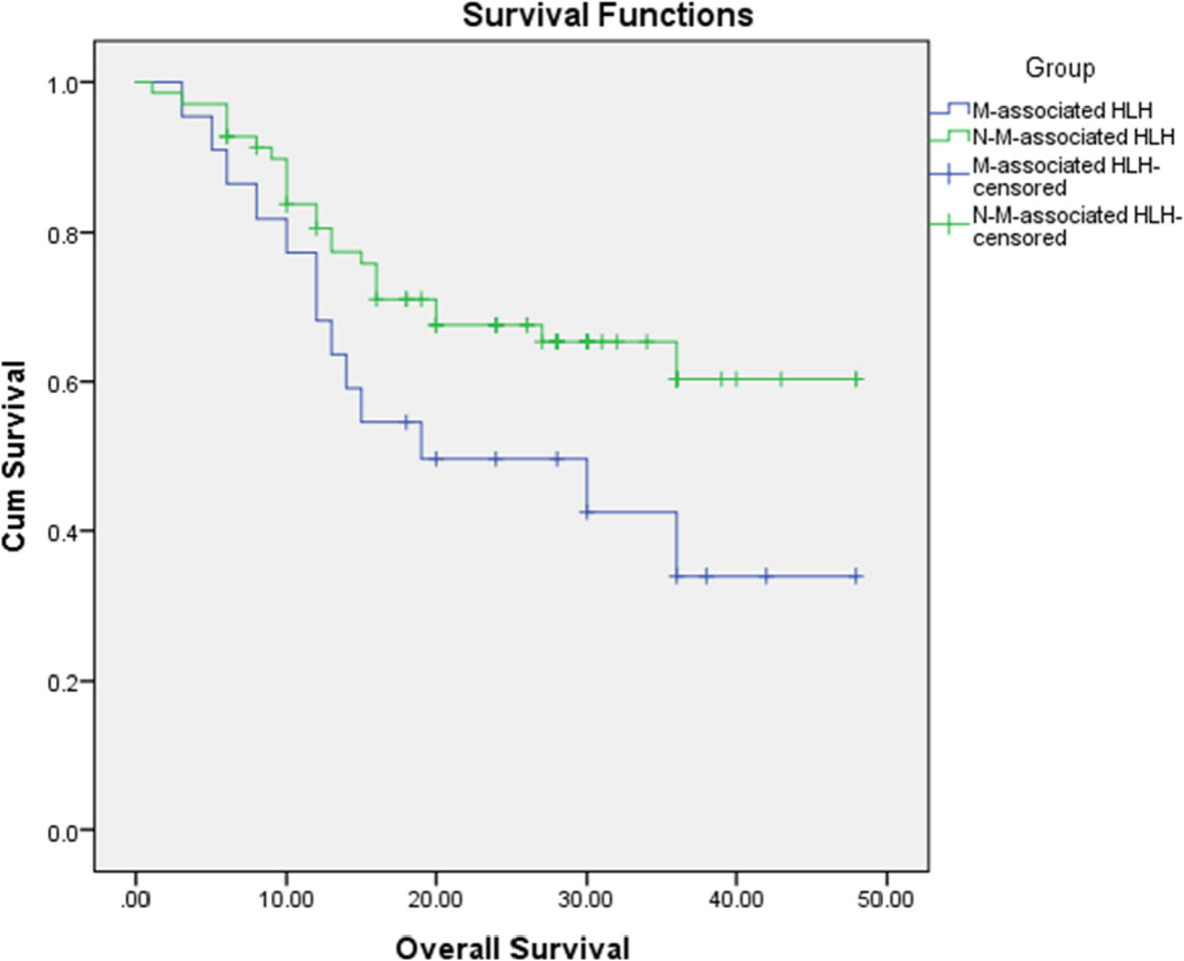
Kaplan–Meier survival of patients with malignancy-associated HLH and non-malignancy-associated HLH

The mean survival time in the malignancy–associated HLH and non-malignancy–associated HLH groups was 26.9 ± 3.82 and 35.03 ± 2.19 months, respectively. The difference in the overall survival time between the two groups was not statistically significant (*p* = 0.055) (Fig. 1).

### Prognostic factors

Correlation analysis of the prognostic factors associated with malignancy-associated HLH showed that Fib and DIC (*r* = 0.446, *p* = 0.037), ferritin and PLT (*r* = 0.516, *p* = 0.014), ALT (*r* = 0.481, *p* = 0.023), albumin and PLT (*r* = 0.483, *p* = 0.023), AST and ALT (*r* = 0.687, *p* = 0.001), and pleural effusion and pneumonia (*r* = 0.399, *p* = 0.041) were correlated. In prognostic analysis, factors with absolute correlation coefficient values exceeding 0.7 (i.e., *r* > 0.7) should not be simultaneously entered. Therefore, all prognostic factors could be included for prognostic analysis.

We conducted univariate analysis to assess the association between prognostic factors and survival time in malignancy-associated HLH (Table 2). DIC score > 5 at diagnosis (*p* = 0.001), albumin < 25 g/L (*p* = 0.000), HGB < 60 g/L (*p* = 0.001), and PLT < 30 × 10^9^/L (*p* = 0.042) were negative prognostic factors for patients with malignancy-associated HLH. Multivariate analysis showed that albumin < 25 g/L (*p* = 0.017), HGB < 60 g/L (*p* = 0.027), and bone marrow hemophagocytosis (*p* = 0.034) correlated with poor prognosis (Table 3). The other factors did not significantly affect survival time in the two groups.

**Table 2:** Results of univariate analysis of prognostic factors of malignancy-associated hemophagocytic lymphohistiocytosis (HLH)

Variable	HR	95% CI	*p* value	*Q* value
Age	0.966	0.873–1.069	0.506	0.843
Gender	0.815	0.418–1.587	0.547	0.841
EBV-positive	0.864	0.437–1.708	0.674	0.963
@Ne	0.949	0.823–1.095	0.476	0.865
HGB (< 60 g/L)	0.968	0.949–0.987	0.001	0.01
Fibrinogen	1.043	0.744–1.462	0.807	1.009
Ferritin	1.000	1.000–1.000	0.878	1.033
LDH	1.000	1.000–1.000	0.469	0.938
DIC	3.045	1.571–5.901	0.001	0.007
Jaundice	1.614	0.838–3.109	0.153	0.612
Bone marrow involvement	0.722	0.375–1.390	0.330	0.733
TG	0.890	0.718–1.103	0.288	0.823
PLT (< 30 × 10^9^/L)	0.993	0.985–1.000	0.042	0.210
Albumin (< 25 g/L)	0.883	0.827–0.943	0.000	0.000
ALT	1.000	1.000–1.000	0.951	1.001
AST	1.000	1.000–1.000	0.213	0.710
Splenomegaly	1.038	0.487–2.210	0.923	1.026
Cholecystitis	1.022	0.360–2.896	0.968	0.968
Pleural effusion	0.876	0.431–1.781	0.714	0.952
Pneumonia	1.389	0.721–2.676	0.325	0.813

*HR* Hazard ratio, *EBV* Epstein–Barr virus, *HGB* Hemoglobin, *LDH* Lactate dehydrogenase, *DIC* Disseminated intravascular coagulation, *TG* Triglycerides, *PLT* Platelets, *ALT* Alanine aminotransferase, *AST* Aspartate aminotransferase, *@Ne* Neutrophils granulocyte

**Table 3:** Results of multivariate analysis of prognostic factors of malignancy-associated hemophagocytic lymphohistiocytosis (HLH)

Variable	HR	95% CI	*p* value
Group	1.893	0.698–5.134	0.210
Age	1.069	0.939–1.219	0.313
Sex	0.855	0.351–2.082	0.730
EBV-positive	0.861	0.375–1.980	0.725
@Ne	0.963	0.789–1.175	0.708
HGB (< 60 g/L)	0.973	0.950–0.997	0.027
Fibrinogen	1.242	0.808–1.910	0.322
Ferritin	1.000	1.000–1.000	0.235
LDH	1.000	1.000–1.000	0.714
DIC	1.745	0.700–4.353	0.232
Jaundice	1.911	0.715–5.105	0.197
Bone marrow involvement	0.374	0.151–0.928	0.034
TG	0.897	0.725–1.110	0.316
PLT (< 30 × 10^9^/L)	1.000	0.992–1.007	0.917
Albumin (< 25 g/L)	0.875	0.783–0.976	0.017
ALT	1.000	0.999–1.001	0.615
AST	1.000	1.000–1.001	0.491
Splenomegaly	0.641	0.238–1.727	0.379
Cholecystitis	1.329	0.381–4.641	0.655
Pleural effusion	0.520	0.189–1.431	0.205
Pneumonia	1.135	0.521–2.476	0.750

*HR* Hazard ratio, *95% CI 95%* confidence interval, *EBV* Epstein–Barr virus, *HGB* Hemoglobin, *LDH* Lactate dehydrogenase, *DIC* Disseminated intravascular coagulation, *TG* Triglycerides, *PLT* Platelets, *ALT* Alanine aminotransferase, *AST* Aspartate aminotransferase, *@Ne* Neutrophils granulocyte

### Role of allogeneic HSCT in malignancy-associated HLH

In our cohort, only five patients (three patients with leukemia; two patients with lymphoma) received allogeneic HSCT. Four patients achieved complete remission after allogeneic HSCT. One patient with HD achieved partial remission, but died of HD relapse, complicated by infection.

## Discussion

We performed a retrospective analysis of 22 children with malignancy-associated HLH and 69 children with non-malignancy–associated HLH at a single institution. The pathogenesis of SHLH remains unclear. Delavigne et al. [6] proposed extended 18-point diagnostic criteria that are more easily and rapidly available in smaller institutions and primary care settings than the HLH-2004 variables. Non-malignancy–associated HLH is common in acute self-limited infectious mononucleosis (IM), rheumatic or autoimmune diseases, immunodeficiency diseases, and CAEV.

EBV is the most frequent antigen activator of SHLH [7]. The pathological changes in IM and CAEV differ. In contrast to B cell infection in IM, CAEV features the proliferation and infection of polyclonal, predominantly non-CD8^+^ (CD4^+^CD8^−^ and CD4^+^CD8^+^) T cells, and CD16^+^ natural killer (NK) lymphocytes [8]. In CAEV, mortality generally results from the subsequent development of HLH and/or T/NK lymphoproliferative neoplasm [9]. The prognosis is poor once CAEV develops into HLH [9]. Chronic granulomatous disease is an inherited disorder of phagocyte nicotinamide adenine dinucleotide phosphate (NADPH) oxidase, which may be associated with HLH [10, 11]. HLH is characterized by impaired function of T cell–mediated inflammation, which is partly regulated by NADPH oxidase. This pathophysiological cooperation may account for the increased severity.

EBV-related HLH is an acquired, infection-related HLH that typically represents a fulminant presentation of acute EBV infection of CD8^+^ T cells, and has a mortality rate of 30–50% [8]. In the present study, 30.4% of the children with non-malignancy–associated HLH were EBV-positive; eight patients (36.4%) with malignancy-associated HLH were positive for EBV infection. Ahn et al. [12] suggested that patients with high EBV DNA viral load have poor prognosis. In the present cohort, a 12-year-old girl with γδ T cell lymphoma relapsed and developed hemophagocytic syndrome after receiving chemotherapy for 6 weeks; she died of severe EBV infection. Strenger et al. [13] found that malignancy-induced HLH concurrent with EBV infection might be a possible trigger in immunocompromised patients.

In children, HLH may be associated with SLE, a systemic autoimmune disorder involving multiple visceral organs. In HLH due to SLE, corticosteroids and immunosuppressive agents, including cyclosporine, cyclophosphamide, intravenous immunoglobulin, and etoposide, have been used with variable success [14].

In the present study, patients with non-malignancy–associated HLH had significantly higher HGB (*p* = 0.004), ALT (*p* = 0.002), and AST (*p* = 0.001) levels than the patients with malignancy-associated HLH. The cause of low HGB in malignancy-associated HLH may be associated with the inhibition of hematopoiesis by malignance and chemotherapy. However, the cause of high ALT and AST levels in non-malignancy-associated HLH is unclear. Damage to liver function is characterized by severe inflammation and immune-mediated organ damage. Inflammatory cell proliferation and infiltration into organs and tissues and uncontrolled hypercytokinemia in non-malignancy–associated HLH may be more obvious than that in malignancy-associated HLH [15].

The difference in survival time was not statistically significant between the two groups (*p* = 0.055). However, previous studies have confirmed that patients with malignancy have worse survival than those without malignancy [16–18]. Celkan et al. [19] reported 54% overall survival in 13 children and adolescents with malignancy-associated HLH. The 13 children included five patients with leukemia; eight patients with rhabdomyosarcoma, neuroblastoma, or lymphoma; and one patient with Langerhans cell histiocytosis. Another study reported that the 2-year survival rate of 25 children with malignancy-associated HLH was 40.9%, and survival was 56% following the acute phase of HLH; a 5-year survival rate of 36% has also been reported [20]. We did not detect significant differences in outcome between malignancy-associated HLH and non-malignancy–associated HLH. Our results show that the low survival rate in the latter group might be due to CAEV, immunodeficiency diseases, and autoimmune diseases, as the prognosis of the abovementioned underlying diseases was poor.

The factors that affect survival are not well known, and there are currently no standard outcome predictors for SHLH. Recent data have shown that ferritin reduction is a prognostic variable for mortality in children with HLH [21]. Following univariate analysis, Park et al. [22] found that serum Fib ≥166 mg/dL at the initial visit was significantly associated with survival time. Low histiocyte proportion in the bone marrow and early initiation of treatment are generally correlated with favorable outcome. However, we did not find any relationship between the decline in serum ferritin, Fib level, and survival. Univariate analysis demonstrated that DIC score > 5 at diagnosis (*p* = 0.001) and albumin < 25 g/L (*p* = 0.000), HGB < 60 g/L (*p* = 0.001), and PLT < 30 × 10^9^/L (*p* = 0.042) were negative prognostic factors for patients with malignancy-associated HLH. The multivariate analysis showed that HGB < 60 g/L (*p* = 0.027), albumin < 25 g/L (*p* = 0.017), and bone marrow hemophagocytosis (*p* = 0.034) were negative prognostic factors for both groups. A previous comprehensive study involving 52 Turkish children with HLH suggested that high DIC score (≥5) and age < 2 years at diagnosis are important risk factors that can increase the mortality risk by twofold [23]. We also found that high DIC score (≥5) and low albumin, HGB, and PLT levels predict poor progress.

Most cases of non-malignancy–associated HLH should be treated aggressively with standard HLH protocols. In particular, the prognosis for such cases has improved dramatically with chemotherapy and immune-modifying agents such as corticosteroids, intravenous immunoglobulins, cyclosporin A, and etoposide.

The treatment of malignancy-associated HLH has not been prospectively studied. In the present study, the malignancy-associated cohort received malignancy-directed treatments, HLH-directed treatments, or a combined approach to overcome HLH. However, we could not determine which approach was superior. In a murine model of familial HLH type 2, mice that received etoposide, cyclophosphamide, or methotrexate survived [24]. Regimens involving the three agents can possibly treat malignancy-associated HLH. Moreover, clinical experience is greatest for etoposide [25]. In malignancy-associated HLH, 13 patients (59.1%) received etoposide as initial therapy for HLH, and six children are alive; the survival rate was 46.2%. Therefore, we found that etoposide is effective.

Stem cell transplantation might be considered consolidation in malignancy-associated HLH. The complete remission rate of malignancy-associated HLH was 90%. Therefore, stem cell transplantation is an effective therapy.

## Conclusions

We analyzed a single center’s experience with malignancy-associated HLH and non-malignancy–associated HLH in children. The etiology of malignancy-associated SHLH is unclear. New research has demonstrated that an underlying polygenic inheritance defect should be suspected in acquired HLH [4]. Prospective studies with a randomized controlled design and involving large cohorts of children are warranted to evaluate the exact incidence, clinical features, and appropriate treatment protocols of this association. Mutation analysis, effective diagnostic criteria, and new targeted treatments for HLH for improving the survival rate of patients with malignancy-associated HLH are necessary.

## Data Availability

Data are available from the authors upon reasonable request.

## Abbreviations

ALL: Acute lymphoblastic leukemia
ALT: Alanine aminotransferase
AML: Acute myeloblastic leukemia
ANC: Absolute neutrophil count
AST: Aspartate aminotransferase
CAEV: Chronic active EBV infection
DIC: Disseminated intravascular coagulation
Fib: Fibrinogen
HD: Hodgkin lymphoma
HLH: Hemophagocytic lymphohistiocytosis
HSCT: Hematopoietic stem cell transplantation
IM: Infectious mononucleosis
NADPH: Nicotinamide adenine dinucleotide phosphate
NK: Natural killer
PLT: Platelet
SHLH: secondary HLH
SLE: systemic lupus erythematosus
